# Adnexal Incarceration in a Posterior Pelvic Peritoneal Defect Associated with Ovarian Torsion: A Case Report

**DOI:** 10.1055/s-0045-1801841

**Published:** 2025-03-11

**Authors:** Lina Qattea, Wafa Alshahrani, Samaher Samer Alouch, Abdulrazzaq Qattea, Wafaa Qatteh, Sami Qattea

**Affiliations:** 1 Department of Obstetrics and Gynecology, King Abdulaziz Medical City, National Guard Health Affairs, Riyadh, Saudi Arabia; 2 College of Medicine at Al Faisal University, Riyadh, Saudi Arabia

**Keywords:** ovarian torsion, pelvic pain, peritoneal defect, surgery

## Abstract

Ovarian torsion is one of the gynecological emergencies and surgical intervention is the standard management for ovarian salvage as well as reveals some unexpected anatomical defects that increase the risk of ovarian torsion. We report a case of a 27-year-old single, nulliparous female taken for diagnostic laparoscopy with suspicion of ovarian torsion. Intraoperatively, we found right adnexa torsion (ovary with tube) along with incarceration of the right ovary and tube into the big peritoneal sac, which was located medial to the right uterosacral ligament. Detorsion and careful pulling of the swollen adnexa were done to the outside of the big peritoneal sac using nontraumatic laparoscopic forceps. The left side was normal with normal left adnexa. The patient had a second laparoscopic look with a plan for peritoneal closure of the big peritoneal defect.

There are two cases which have previously reported ovarian incarceration, but were not associated with ovarian torsion, and did not involve peritoneal closure unlike our report of ovarian detorsion and peritoneal defect closure. The etiology of this condition is thought to be likely congenital as there were no other visible etiologies like pelvic trauma, previous surgery, pelvic inflammatory disease, or endometriosis.

Our clinical assessment suggests that a peritoneal sac can enhance the course of ovarian torsion to involve ischemic changes of the ovary when trapped inside of it, by its prevention of spontaneous detorsion. This condition will result in the compression of the cells between the sac wall and subsequent tissue edema enhancing the ischemic effect.

## Introduction

Surgical evaluation for presentation of acute pelvic pain is often necessary to explore its etiology as well as its risk factors in females of reproductive age. 1 Laparoscopy plays a role in diagnosis and treatment in emergency gynecology cases as it confirms ovarian torsion and could show the possible cause and risk of twisted ovary in women. 2 Some underlying causes are rare and unexpected, such as a peritoneal hernia or a defect in which the ovary and adnexa can fall and become incarcerated along with the torsion. 1

Some of the congenital causes are Mullerian duct anomalies such as Mayer–Rokitansky–Kuster–Hauser (MRKH) syndrome. In patient diagnosed with MRKH syndrome, congenital absence of the upper two-thirds of the vagina, the cervix, and hypoplasia, or complete aplasia of the uterus. 3 In such patients, the ovaries lack the support of the utero-ovarian ligament, and are only supported by the infundibulopelvic ligament, making the ovaries highly mobile and more suspectable to ovarian torsion. 4 Due to the absence of the uterus, the ovaries are only supported by the infundibulopelvic ligament, which provides little fixation to the pelvic side wall, making ovarian torsion more common in such cases. Ovarian torsion is more common in patients with anatomical variation, including those with ovarian cyst or anatomical agenesis, such as patients with MRKH in which the torsion might include the fallopian tubes with or without the ovary. 5

In general, the presence of incarceration with ovarian torsion plays a role in enhancing the ischemic changes in ovarian tissue by reducing the blood supply and leading to venous congestion, ischemia, and eventually necrosis, making prompt diagnosis crucial. Incarcerated adnexa is rare and there are only two cases reported to date. 1 2 We present a case of an incarcerated ovary combined with ovarian torsion and ischemic change. This case was followed up by a laparoscopic second-look re-exploration procedure and management of the identified peritoneal defect.

## Case Report

A 27-year-old, single, nulliparous female, not known to have any medical conditions, with a surgical history of a gastric sleeve, presented to the emergency room with severe right lower quadrant pain for 1 day. The pain was associated with nausea, however, without any episodes of vomiting. The patient denies any fevers, abnormal vaginal discharges, dysuria, or changes in bowel habits. Her last menstrual cycle was 2 weeks prior to presentation. She had no history of chronic pelvic pain or secondary dysmenorrhea.

Upon examination, the patient was visibly in distress and crying out in pain. Laboratory tests showed a negative urine pregnancy test, a white blood cell count of 6.34 × 10 ^9^ cells/L, with 71% neutrophils, a hemoglobin level of 106 g/L, and a platelet count of 284 × 10 ^9^ cells/L. The lactic acid level was 2.32. A formal gynecological transabdominal ultrasound was performed, which showed an enlarged right ovary in size with absence of blood flow.

**Fig. 1:**
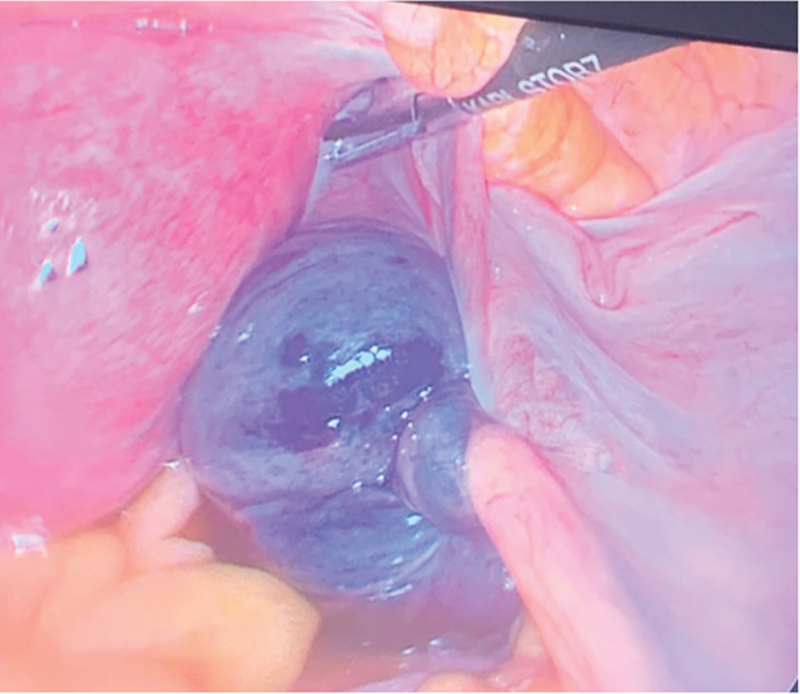
Right adnexa torsion with ovarian ischemic changes.

**Fig. 2:**
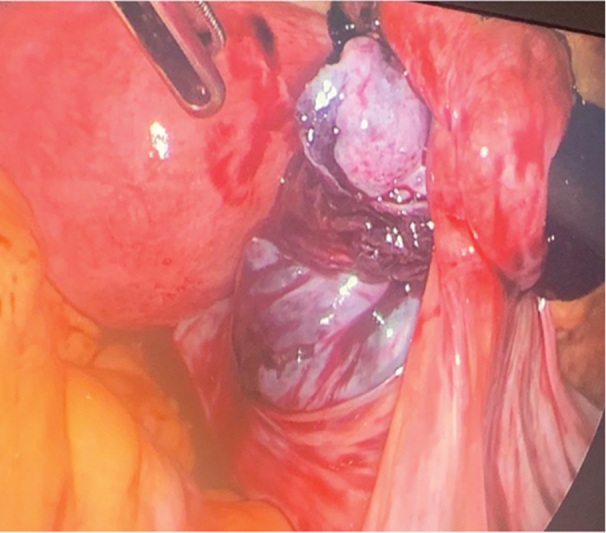
Right ovary incarcerated inside the peritoneal pouch.

**Fig. 3:**
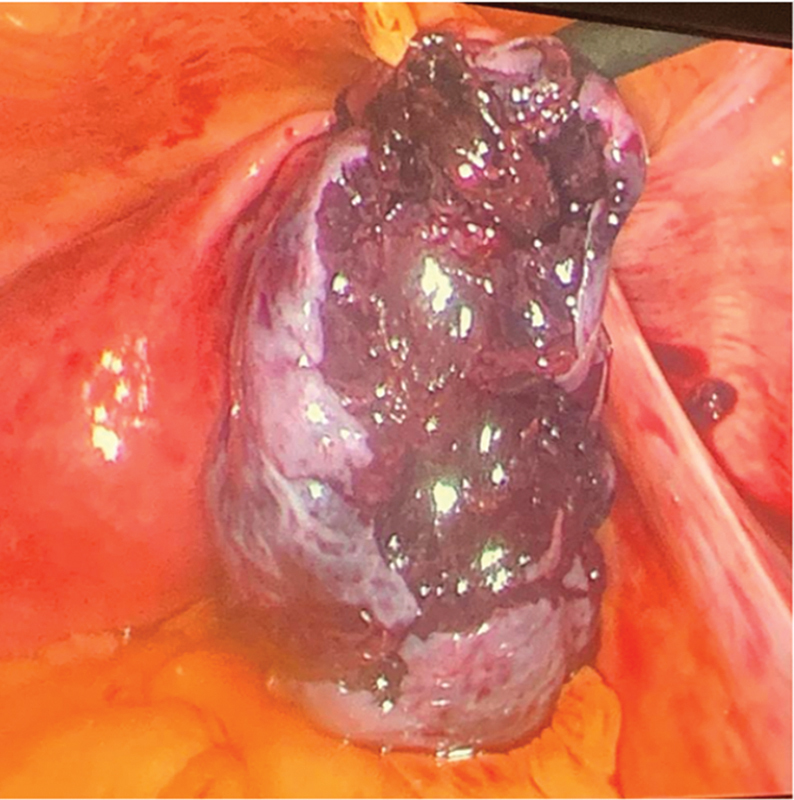
Right ovary completely extracted.

**Fig. 4:**
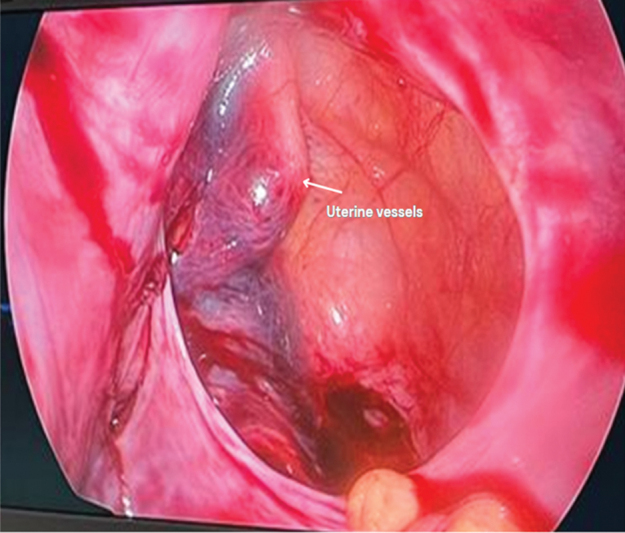
Appearance of uterine vessel's peritoneal pouch.

**Fig. 5:**
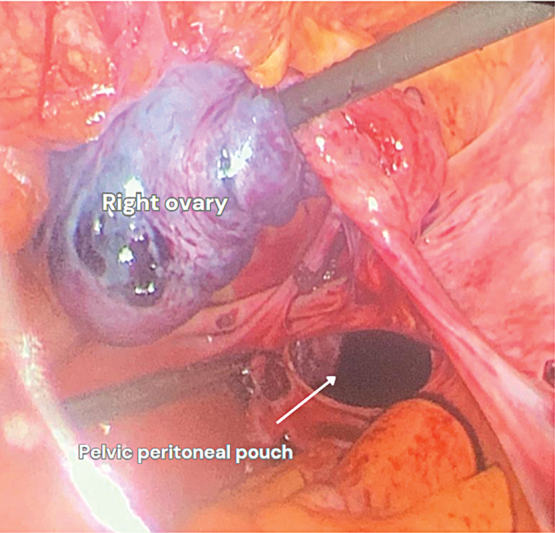
Pelvic retroperitoneal pouch.

The patient was consented and taken to the operating room and a diagnostic laparoscopy was started. Upon visualization, the right ovary and the fallopian tube were noted to be torsed four times ( Fig. 1 ). The right ovary was found to be impacted inside a pelvic peritoneal pouch above the right uterosacral ligament ( Fig. 2 ). The ovary was noted to be enlarged and bluish in color. We started by removing the ovary from the pouch gently using atraumatic graspers, gradually pulling the ovary, which was very fragile ( Fig. 3 ). Once the ovary was completely out, we began detorsion of the right adnexa, ovary, and tube. The inspected peritoneal pouch was found to be 8 × 6 cm, rounded in shape, with visible uterine vessels inside in the medial pouch wall ( Figs. 4 and 5 ). There were no other visible structures or any abnormalities like an endometriotic patch. The left adnexa tube and ovary were inspected and appeared normal with no peritoneal defect in the left side. After inspecting the left adnexa, our gaze was shifted back to the right ovary, which began to regain its normal color after few minutes of detorsion. No ovariopexy was performed. We ended the procedure with a plan for a second laparoscopic look for closure of the identified defect as the pouch tissue was edematous and fragile at the time and would make the suturing difficult. The patient tolerated the surgery, and she was discharged in stable condition.

**Fig. 6:**
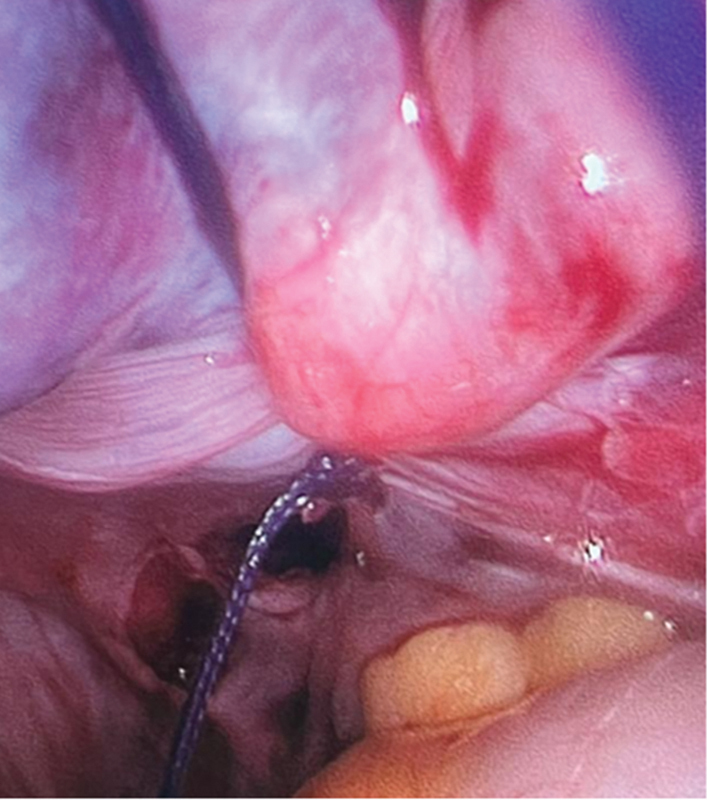
Closing peritoneal pouch with interrupted suture.

Four weeks post-op, the patient was taken for a second look laparoscopic surgery for a better closure of the identified defect. Intraoperatively, both ovaries and tubes looked normal. The right tube and ovary had regained their normal structure, size, and color ( Fig. 6 ). The defect seen previously in the right side of the posterior pelvic peritoneum was smaller than before, measuring around 5 × 6 cm. The defect was closed by approximating the edges and suturing using an interpreted 0 Vicryl stitch interrupted after refreshing the edges around. The closure was started from medial to the lateral, 1 to 2 cm left over as opening in the lateral side. The procedure ended with no complications. The patient tolerated the second surgery and was discharged from the hospital on post-op day 1 with no complications. The patient was then seen in the clinic 3 weeks postoperation for a follow-up, which was reassuring. The patient claimed to have no more pain and that her menstrual cycle had since resumed regularly.

## Discussion

The diagnosis of ovarian torsion can be challenging due to its vague symptoms upon presentation. Abdominal pain is usually reported to be the most common symptoms followed by nausea and vomiting, which present in 85 to 90% of the cases. 6 7 Ultrasound is the first imaging modality that is used for differential diagnosis in patients with acute abdominal or pelvic pain. B-mode ultrasonography is used to assess the ovarian morphology while color Doppler ultrasonography (CDU) is used to evaluate the blood flow to the ovaries. 8 It has been proposed that the decrease of blood flow to the ovaries is highly suggestive of ovarian torsion. However, recent studies have argued about the reliability of color Doppler in the diagnosis of ovarian torsion due to its low sensitivity. It was reported in a study that the CDU sensitivity in the diagnosis of ovarian torsion reaches 43% with a specificity of 91.7%, while the negative predictive value reached 71% and the positive predictive value reached a 78 to 100%. 9 10 Such findings have implied that it is not recommended to rule out the diagnosis of ovarian torsion based on normal CDU. Furthermore, the American College of Obstetricians and Gynecologists suggested not to use CDU for the diagnosis of ovarian torsion and rely more on the clinical judgment. 11 The pathophysiology of ovarian torsion is still not clear. A recent published article by Carugno stated that an association was noted between leukocytosis and absent blood flow in patients with ovarian torsion. It is believed that the etiology behind having a high inflammatory marker is a response to necrosis of the tissue during the ovarian torsion. Therefore, it is hypothesized that the presence of no blood flow and high inflammatory markers is an indicator that ischemia started to occur and ovarian torsion is more. 12

Torsion affects the right side of the body more frequently than the left, with a 3:2 ratio, as explained earlier. 13 The first step during surgical approach is to detorse the adnexa after the laparoscopic ports have been inserted. Untwisting the torsed adnexa was originally thought to cause vascular emboli, therefore most torsion was treated by removing the adnexa without untwisting it. 14 However, this has been proven to be false. 15 Even if the ovary seems blue and dusky at first glance, after only 6 weeks, most ovaries, ∼90%, show normal follicular development on ultrasound, normal Doppler flow, and normal gross appearance. 16

Pelvic peritoneal defect is a rare occurrence, and can be congenital or iatrogenic. 2 Dysfunctional embryonic uterine development is believed to be the cause of congenital peritoneal defect. It is believed that during embryonic development, the remnant of the Mesonephric or Mullerian duct forms a cyst in the broad ligament which later can rupture and lead to a defect in the peritoneum. Iatrogenic pelvic peritoneal defects are typically seen in endometriosis, prior surgery, intraperitoneal inflammation, obstetric or abdominal trauma, and iatrogenic operations as the most common predisposing factors of peritoneal defect. 17 Another etiology is Allen–Masters syndrome, a syndrome which is associated with laceration to the uterine-supportive ligaments following traumatic delivery. 18

This differential makes the defect in this case more likely to be congenital in nature due to the absence of a history of pelvic surgery, nulliparity, the absence of prior sexual intercourse, and any history of sexually transmitted diseases, along with the lack of signs of endometriosis. Together, these findings suggest that our patient's peritoneal defect was likely congenital. However, the etiology of peritoneal defect is idiopathic. In 0.5% of regular biopsy specimens, congenital defects were discovered. 19 Ovarian incarceration into a pelvic peritoneal defect is a rare phenomenon, and there are only few published case reports to guide management of the defect. 1

Literature review of the pelvic peritoneal defect with incarcerated ovary showed two cases published describing ovarian incarceration into a posterior pelvic peritoneal defect like our case, by Kataoka et al in 2009 and by Jackson et al in 2015. 1 2 This is a third case describing an incarcerated ovary. The previous two published cases were on the right adnexa pelvic side with similar clinical presentations and normal ovarian tissue on laparoscopy with no evidence of ischemic changes such as abnormal discoloration or evidence of ovarian torsion. Our case was associated with ovarian torsion four times, and there were ischemic changes of the affected ovary which made the removal of the incarcerated ovary from the defect more challenging considering the fragility of the ovarian tissue.

In this case, we chose to close the peritoneal defect compared with the two published studies, which left the defect with no suturing. Surgical repair of the peritoneal defect is still controversial, with literature review revealing lack of consensus. 1 20 21 We closed the defect because we believed it may prevent the recurrence of an incarcerated ovary that would otherwise increase the risk of ovarian ischemia with ovarian torsion. On the other hand, most reports of a peritoneal defect support not closing the peritoneum to prevent peritoneal tension and the risk of vascular injury. 1 Other methods for closing peritoneal defects include using an omental ﬂap, mesh, or a space-occupying compound. 5 22 More cases are required to be collected and reported to support or go against the defect closure, weighing the benefits and risks of such intervention and the risk of re-occurrence.

## Conclusion

In conclusion, pelvic peritoneal defect is considered to be a rare phenomenon. More case reports and long-term sequels are required to identify whether the defect is associated with enhancing the ovarian ischemia with torsion. Closure of the peritoneal defect is still controversial and requires more studies for an expert consensus on the best approach to those findings.
